# Screening for bipolar disorder in individuals engaged in artistic training

**DOI:** 10.1192/j.eurpsy.2026.11842

**Published:** 2026-07-31

**Authors:** H. Mezghani, N. Regaieg, F. Guermazi, R. Masmoudi, I. Feki, J. Masmoudi, I. Baati

**Affiliations:** Psychiatry A Department, Hedi Chaker University Hospital, Sfax, Tunisia

## Abstract

**Introduction:**

The emotional reactivity and affective instability that characterize the artistic lifestyle could fall within the spectrum of bipolar disorder. However, the boundaries between intense creative functioning and mood fragility are of greater interest.

**Objectives:**

The objective of this study is to screen for bipolar disorder in a population of students at the Institute of Arts and Crafts of Sfax, Tunisia.

**Methods:**

Our study was cross-sectional and descriptive, conducted between February 1^st^ and March 5^th^, 2025, among students at the Institute of Arts and Crafts in Sfax. Students who completed an anonymous self-administered questionnaire containing sociodemographic, clinical, and other data were included in the study. A psychometric scale in its Arabic version: The Mood Disorder Questionnaire (MDQ), was used for screening for bipolar disorder.

**Results:**

The study included 60 students with a sex ratio of 0.6. The median age was 20 years [19-22 years].

Participants had a family history of mood disorders in 20% of cases and of suicide attempts in 16.7% of cases. Regarding individual history, 7% of students reported a history of depressive mood disorder occurring before the age of 25 years. A history of suicidal ideation or suicide attempts was found in 18.3% of participants. Furthermore, 25% of students reported using psychoactive substances.

In connection with their artistic lifestyle, 50% of students felt it impacted their sleep and eating routines, and 33.3% experienced feelings of loneliness due to their artistic work. Furthermore, 66.7% believed that the artistic field could exacerbate emotional difficulties, and 41.7% reported making impulsive decisions during periods of intense emotion.

Finally, screening for bipolar traits using the MDQ revealed a positive score in 28.3% of students (17 cases).

**Conclusions:**

The relationship between an artistic lifestyle and bipolar vulnerability remains complex. Identifying distinct at-risk profiles facilitates the development of more tailored support and prevention strategies for these students.

**Disclosure of Interest:**

None Declared

